# Bioactive glass S53P4 eradicates *Staphylococcus aureus* in biofilm/planktonic states *in vitro*

**DOI:** 10.1080/03009734.2020.1765908

**Published:** 2020-06-18

**Authors:** Torstein Grønseth, Lene K. Vestby, Live L. Nesse, Magnus von Unge, Juha T. Silvola

**Affiliations:** a Institute for Clinical Medicine, University of Oslo, Oslo, Norway;; b Department of Otolaryngology, Head and Neck Surgery, Oslo University Hospital, Oslo, Norway;; c Department of Analysis and Diagnostic, Norwegian Veterinary Institute, Oslo, Norway;; d Department of Animal Health and Food Safety, Norwegian Veterinary Institute, Oslo, Norway;; e Department of Otolaryngology, Head and Neck Surgery, Akershus University Hospital, Akershus and Oslo, Norway;; f Center for Clinical Research, Uppsala University, Västerås, Sweden

**Keywords:** BAG, biofilm, confocal laser scanning microscopy, *Staphylococcus aureus*

## Abstract

**Background:** Increasing antimicrobial resistance to antibiotics is a substantial health threat. Bioactive glass S53P4 (BAG) has an antimicrobial effect that can reduce the use of antibiotics. The aim of this study was to evaluate the antimicrobial efficacy of BAG *in vitro* on staphylococci in biofilm and in planktonic form. Secondary aims were to investigate whether supernatant fluid primed from BAG retains the antibacterial capacity and if ciprofloxacin enhances the effect.

**Methods:** BAG-S53P4 granules, <45 µm, primed in tryptic soy broth (TSB) were investigated with granules present in TSB (100 mg/mL) and after removal of granules (100, 200, and 400 mg/mL). The efficacy of BAG to eradicate *Staphylococcus aureus* biofilm *in vitro* was tested using 10 different clinical strains and 1 reference strain in three test systems: the biofilm-oriented antiseptic test based on metabolic activity, the biofilm bactericidal test based on culturing surviving bacteria, and confocal laser scanning microscopy (CLSM) combined with LIVE/DEAD staining.

**Results*:*** Exposure to 48 h primed BAG granules (100 mg/mL) produced bactericidal effects in 11/11 strains (*p* = 0.001), and CLSM showed reduction of viable bacteria in biofilm (*p* = 0.001). Supernatant primed 14 days, 400 mg/mL, reduced metabolic activity (*p* < 0.001), showed bactericidal effects for 11/11 strains (*p* = 0.001), and CLSM showed fewer viable bacteria (*p* = 0.001). The supernatant primed for 48 h, or in concentrations lower than 400 mg/mL at 14 days, did not completely eradicate biofilm.

**Conclusion:** Direct exposure to BAG granules, or primed supernatant fluid, effectively eradicated *S. aureus* in biofilm. The anti-biofilm effect is time- and concentration-dependent. When BAG had reached its full antimicrobial effect, ciprofloxacin had no additional effect.

## Introduction

Bacteria can alternate between a planktonic and a biofilm state. As biofilm, bacteria are attached to a substratum, or to each other, and are embedded in a self-produced extracellular polymeric substance. Since the 1970s, when Nils Høiby observed a link between the aetiology of a persistent infection and aggregates of bacteria in cystic fibrosis patients (1), biofilm infections have become increasingly recognised in chronic infections. A common pathogen is *Staphylococcus aureus*, a potent biofilm producer (2–6). Bacteria living in biofilm display an altered antimicrobial resistance phenotype through a lower metabolic rate and a reduced rate of cell division (7,8). In addition, the biofilm can act as a diffusion barrier, and also cause deactivation of antimicrobial substances (9–11). As a result, the minimum inhibitory concentration of antimicrobial compounds on bacteria in biofilm can reach 500–1000 times that of their planktonic counterparts (9,12). Mature biofilm can further shed off planktonic bacteria or micro-colonies that may travel to other parts of the body, causing relapsing infections (10).

Because of these biofilm defense mechanisms and growing antimicrobial resistance (13,14), we need innovative new treatments in addition to conventional antimicrobial treatment. One such option is bioactive glass (BAG), a synthetic silica-based material. BAG gradually releases ions from the granules’ surfaces when in contact with biological fluids. This leads to an increase in osmotic pressure and pH, thus making the surrounding environment unsuitable for microbial growth (15–17). There are several types of BAG with differing antibacterial activity, one of which, BAG-S53P4, has been well studied (15,18). In clinical practice, BAG has been used to fill bone cavities (19) and treat osteomyelitis in orthopaedic surgery (20), chronic frontal sinusitis during frontal sinus obliteration (21), and chronic otitis media during mastoid obliteration (22,23). The diameter of the granules is usually between 0.5 mm and 3.15 mm.

The main aim of the study was to evaluate the antimicrobial efficacy of BAG-S53P4 *in vitro* on clinical strains of *S. aureus* in biofilm and planktonic form. The sub-aims were to investigate whether the supernatant fluid primed from BAG retained anti-biofilm properties after removal of the granules, and to evaluate whether addition of ciprofloxacin causes an increased anti-biofilm effect.

## Methods

### Study design

Ten clinical strains of *S. aureus* and one reference collection strain were first studied to verify their biofilm-producing capacity. The 11 bacterial strains were then tested in a planktonic state for sensitivity to BAG (broth dilution test) and ciprofloxacin (disc diffusion test). If a strain was not sensitive in the planktonic state, it was excluded from further tests in the biofilm form. The antimicrobial effect on bacteria living in biofilm *in vitro* was then tested using three different methods: biofilm-oriented antiseptic test (BOAT); biofilm bactericidal test; and confocal laser scanning microscopy (CLSM).

### Bacterial strain collection and verification

Ten clinical strains of *S. aureus* were collected from 10 randomly selected unique individuals with draining ears at Oslo University Hospital, in a period from April to October 2014. *S. aureus* ATCC 29213, a previously described strain known for its biofilm-producing capabilities, was used as a positive control (24,25). The strains from the draining ears were obtained by using an otomicroscope and a sterile swab (VWR transport swabs, Copan, Breschia, Italy). The identification and antibiotic susceptibility testing did not reveal any MRSA strains (MALDI-TOF-MS, Bruker Daltonik GmbH, Bremen, Germany; VITEK^®^ 2, bioMérieux S.A. France). The bacteria were stored in a freezing broth at −70 °C (Frysebuljong, Oslo University Hospital, Oslo, Norway). Before the tests, each strain was plated onto blood agar plates (Blod agar plater, Oslo University Hospital, Oslo, Norway) for amplification and verification of purity. The blood agar plates were incubated for 24 h at 37 ± 1 °C.

### Test substance

Our study evaluated the antimicrobial efficacy of smaller granules of BAG-S53P4, <45 µm, (donated by BonAlive Biomaterials, Turku, Finland), which have previously been shown to have greater antimicrobial activity compared with larger-sized granules (16). The concentrations and exposure times used are shown in Table 1. The concentrations were selected based on our broth dilution test and on previous studies (26).

**Table 1.** Test substance and exposure time.

Test substance	Priming time
BAG-S53P4 < 45 µm, TSB with 1% glucose/1% NaCl	
Granule test	
BAG - TSB, 100 mg/mL	2 days
Supernatant test	
BAG - TSB, 100 mg/mL	2 days
BAG - TSB, 200 mg/mL	2 days
BAG - TSB, 400 mg/mL	2 days
BAG - TSB, 100 mg/mL	14 days
BAG - TSB, 200 mg/mL	14 days
BAG - TSB, 400 mg/mL	14 days
BAG - TSB, 100 mg/mL with ciprofloxacin 5 µg/mL	2 days
BAG - TSB, 200 mg/mL with ciprofloxacin 5 µg/mL	2 days
BAG - TSB, 400 mg/mL with ciprofloxacin 5 µg/mL	2 days
BAG - TSB, 100 mg/mL with ciprofloxacin 5 µg/mL	14 days
BAG - TSB, 200 mg/mL with ciprofloxacin 5 µg/mL	14 days
BAG - TSB, 400 mg/mL with ciprofloxacin 5 µg/mL	14 days
pH-adjusted (NaOH) TSB with 1% glucose/1% NaCl	
pH-adjusted TSB, pH 7.0	
pH-adjusted TSB, pH 9.7	
pH-adjusted TSB, pH 10.7	
pH-adjusted TSB, pH 11.7	

All TSB with 1% glucose/1% NaCl solutions were from the Norwegian Veterinary Institute, Oslo, Norway.

BAG: bioactive glass; TSB: tryptic soy broth.

The test substance was obtained by adding BAG-S53P4 < 45 µm to tryptic soy broth (TSB) with 1% glucose/1% NaCl, pH 7.0, and vortexed (60 s) to ensure that all granules were in contact with the broth. The test substances were placed at room temperature prior to the experiment. One test substance was prepared for 48 h, and one 14 days prior to the test day. The time that the TSB was exposed to the BAG granules prior to test day is called priming time.

On the test day, the 48-h solutions were further divided into one group with granules (Granule contact test) and one group with only the supernatant (Supernatant test). The 14-day solutions were only tested in the supernatant form (Supernatant test) (Table 1).

#### Granule contact test

Only 100 mg/mL BAG primed for 48 h was tested because of its complete eradication of biofilm at this concentration.

#### Supernatant test

BAG granules were removed from the test solutions, and only the supernatant was used for further testing. It was divided into two sub-tests: i) addition of ciprofloxacin; and ii) without added ciprofloxacin. The concentration of ciprofloxacin was 0.5 µg/mL, based on a previously published study on the expected concentration of ciprofloxacin in the middle ear mucosa (27). No comparisons were made between the efficacy of BAG + ciprofloxacin and BAG alone for the concentrations of 14-day primed 400 mg/mL and 200 mg/mL. The reason is that the bactericidal test for BAG alone showed 0 strains (400 mg/mL) and 3 strains (200 mg/mL) surviving of the total 11 strains.

TSB (with 1% glucose/1% NaCl) broth was pH-adjusted with NaOH, to pH 9.7, 10.7, and 11.7 and used as controls to similar pH-level BAG. The pH was chosen as a result of the highest measured pH from the BAG in TSB for 14 days. All pH measurements used a pH metre (inoLab^®^pH 7310 P, WTW GmbH, Weilheim, Germany).

### Disc diffusion susceptibility testing of ciprofloxacin

Each of the strains of the *S. aureus* was tested in its planktonic state to evaluate the efficacy of ciprofloxacin by a disc diffusion test according to the EUCAST disc diffusion method, version 5. Single colonies from a fresh overnight bacterial culture on blood agar were collected and transferred into sterile saline. The suspension was measured to McFarland 0.5 and the spread on Müller Hinton agar plates using an automated plate spreader. Diffusion discs (CIP 5, Sensi-Disc^TM^, BBL^TM^, Discs, Becton, Dickinson and Company, Sparks, MD, USA) with ciprofloxacin 5 µg were applied to the agar plates. Inhibition zones were evaluated with callipers after 18 h of incubation at 36 ± 1 °C.

### Broth dilution test for planktonic bacteria

A modified broth dilution test was employed to evaluate the efficacy of BAG on planktonic-state bacterial strains (28). First, 48-h suspensions of TSB with BAG were made at concentrations of 50, 100, 200, and 400 mg/mL, separately. Two test sets were prepared for the granule contact and supernatant test. In the granule test, further tests were carried out with the BAG granules in the test solution. In the supernatant test, the supernatant of the primed fluid was pipetted into new sterile tubes without the granules for further testing.

### Bacterial preparation

McFarland standard 0.5 turbidity suspensions of 1 × 108 colony-forming units (cfu)/mL in sterile saline were prepared from 24-h agar incubations of pure cultures from each bacterial strain. The McFarland 0.5 solutions were then diluted in sterile 48-h primed test solution as described above to reach the standard inoculum of 5 × 105 cfu/mL. In the granule test, the supernatant fluid was first moved into new test tubes. In the new test tubes the desired inoculum concentration was reached. Thereafter the supernatant with the inoculum was transferred back to the original test tubes with the BAG. In the supernatant test, the supernatant of the primed fluid was pipetted into new sterile tubes without granules. From the supernatant the described inoculum concentration was prepared, and 200 µL of bacterial inoculum with different primed supernatant concentrations was transferred in triplets to a sterile 96-well microtiter plate (Nunclon Delta Surface, Thermo Fisher Scientific, Roskilde, Denmark). Prepared samples were used within 30 min of preparation. Sterility control with broth only and each concentration of TSB/BAG were included in all experiments. Each bacterial strain was grown in TSB to provide a positive growth control. The test tubes for the granule test and the micro-plates for the supernatant test were kept at 35 ± 1 °C for about 20 ± 0.5 h before being visually evaluated for growth or no growth (28).

### Biofilm assay

The ability of the *S. aureus* strains to form biofilm was tested in a 96-well microtiter plate (Nunclon Delta Surface) according to a previously published method (29). One colony of each bacterial strain was inoculated in 5 ml of TSB, which was cultured overnight at 37 ± 1 °C. The next day, 180 µL of TSB with 1% glucose/1% NaCl was transferred to each of the wells on the microtiter plate, except for the first three blank control wells, where 200 µL was transferred. The overnight cultures were then vortexed for 40 s, and 20 µL was transferred to all the wells, except for the blank control. Each strain of the *S. aureus* was tested in three parallel wells. The microtiter plate was incubated at 37 ± 1 °C for 48 h. The wells were then washed three times with 220 µL of tap water and left to dry at room temperature for 30 min. After drying, 220 µL of crystal violet (1% solution, Sigma Aldrich, St Louis, MO, USA) was added and incubated for 30 min. The wells were washed five times with 220 µL of tap water. To extract the crystal violet from the biofilm, 220 µL of ethanol:acetone (70:30 w:w) was added. The results were calculated by measuring the optical density at 570 nm (Siemens BEP 2000 Advance, Germany). The experiment was repeated three times.

### Biofilm-oriented antiseptics test (BOAT)

In the presence of viable metabolically active bacteria, tetrazolium chloride (TTC) is reduced from a colourless compound to red formazan, which correlates to the number of viable cells (30–32), utilised in the BOAT method (33).

Ten clinical strains of *S. aureus* and the reference collection strain *S. aureus* ATCC 29213 were tested. The same 96-well microtiter plate was used as in the biofilm assay and the biofilm was produced as described above with 12 parallel wells for each strain. After 48 h of incubation, the wells were washed with 220 µL sterile 0.85% NaCl, before adding the active or control substances. The microtiter plate was incubated at 37 ± 1 °C for 24 h. For each strain, three parallel wells were exposed to BAG at concentrations of 400 mg/mL, 200 mg/mL, and 100 mg/mL. Three wells were used as controls. The content in all wells was then removed and Dey Engley neutralising broth was added for 5 min. The wells were filled with 200 µL of TSB:TTC in the ratio of 20:1. The microtiter plate was incubated at 37 ± 1 °C for 7.5 h. The results were evaluated visually by colour change and measured calorimetrically. The amount of formazan produced was calculated calorimetrically by measuring the optical density at 492 nm (Siemens BEP 2000 Advance, Germany). The experiment was repeated three times (34).

### Biofilm bactericidal test

To confirm the eradication effects of BAG solutions on staphylococcus biofilm, a model described by T. Mah was used, modified for staphylococci (33). All 11 strains were tested. The first steps of establishing a biofilm, and applying the test solution, sterile 0.85% NaCl, and neutralising broth were identical to those of the BOAT method described above and as described in our previous study (34). However, instead of then adding TSB:TTC, 200 ml of TSB was added to each well and incubated at 37 ± 1 °C for 24 h. Of the overnight culture 5 ml was transferred from each well onto a blood agar plate and incubated at 37 ± 1 °C for 24 h before the results were evaluated visually. If there was no growth, the tested substance was considered bactericidal. The experiment was repeated three times.

### Confocal laser scanning microscopy (CLSM)

The first steps of making biofilm, applying test solutions, and adding neutralising broth were identical to those of the BOAT method described above, except that a Lab-Tek II Chambered Coverglass with cover 8-well (Thermo Fisher Scientific, Inc., Waltham, MA, USA) was used instead of a microtiter plate. The slides were stained with Filmtracer™ LIVE/DEAD^®^ Biofilm Viability Kit, (Molecular Probes, Thermo Fisher Scientific, Inc., Waltham, MA, USA) according to the manufacturer’s specifications. Images of the stained biofilm were generated on a CLSM (Zeiss LSM710, Germany). A 488 nm argon laser line was used for the SYTO^®^9 and a 561 nm DPSS laser line for the propidium iodide. The ratio of dead or dying cells to the total number of cells in the biofilm was determined by ImageJ software (open source, public domain). The experiment was repeated twice.

### Statistical analysis

Statistical analyses were performed using SPSS statistical software (release 25.0 SPSS Inc., Chicago, Il, USA, and STATA MP/15.1, StataCorp, College Station, TX, USA). *p* < 0.05 was considered to be statistically significant.

In the BOAT, paired *t* test was performed to identify any significant differences between the test substance groups compared to the controls. In the bactericidal test, a McNemar asymptotic test (using STATA MP/15.1) was performed to identify any significant differences between the test substance groups compared to the controls.

In CLSM, significant difference between the treated groups and control was calculated by paired *t* test.

### Approval

The collection of specimens from human subjects was approved by the regional ethical committee (2014/1956, REK sør-øst C).

## Results

### Disc diffusion susceptibility testing of ciprofloxacin

Ciprofloxacin showed clear inhibition zones ≥21 mm in the disc diffusion tests, indicating sensitive bacteria for all strains, except strain 3, which was not used for further testing with ciprofloxacin.

### Broth dilution test for planktonic bacterial strains

A BAG concentration of 50 mg/mL showed growth for all strains (11/11) in both the granule and the supernatant tests. In the test with BAG granules, 100 mg/mL, there was no growth for any strains (*p* = 0.001), but the supernatant test, 100 mg/mL, showed growth in 9/11 strains (*p* = 0.160). The concentrations of 200 and 400 mg/mL of BAG showed no growth for any of strains (*p* = 0.001) in either test.

### Biofilm assay

All strains of staphylococci grew biofilm within 48 h in 96-well microtiter plates. Mean optical density ±1 SD of biofilm produced by 11 *S. aureus* stains was 1.864 ± 0.945. The minimum optical density was 0.851, and the maximum optical density was 3.00.

### Biofilm-oriented antiseptics test (BOAT)

**Figure 1.**
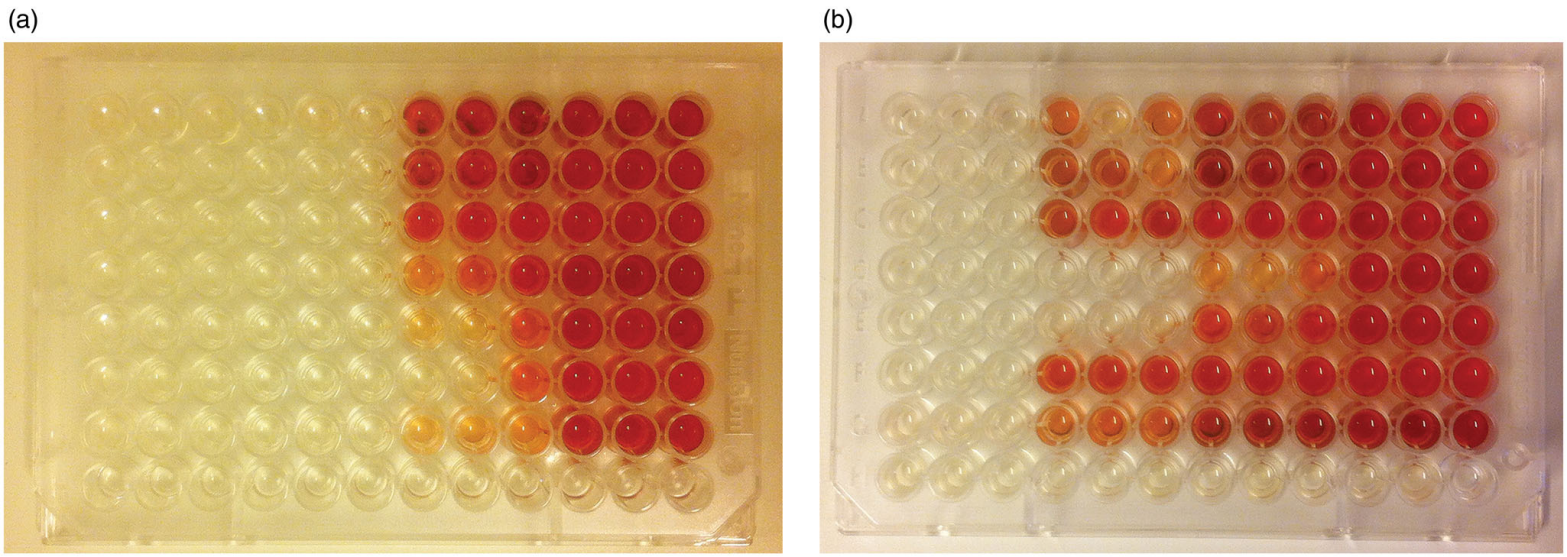
Biofilm-oriented antiseptics test (BOAT). Supernatant test: BAG 14-day priming time (a); BAG 2-day priming time (b). BAG concentration from left to right is 400 mg/mL, 200 mg/mL, 100 mg/mL, and 0 mg/mL; 3 wells per concentration. Rows 1–7: strains 1–7; row 8: negative control. Red formazan is a sign of viable cells. (96-well microtiter plate; Nunclon Delta Surface, Thermo Fischer Scientific).

BOAT was only performed in supernatants, since it was impossible to remove the BAG granules before measuring optical density, thus giving a misleading optical density. The reduction in optical density as an indirect measure of metabolic activity was significant for all concentrations of the test solutions primed for 14 days. After 48 h of priming only the 400 mg/mL concentration showed a significant reduction (Table 2). The optical densities in the 14-day primed test at 400 mg/mL and 200 mg/mL concentrations were similar to the blank control, while the other concentrations and durations showed some metabolically active bacteria in biofilm (Table 2). This was also observed visually, where all wells treated with the 400 mg/mL concentration for 14 days appeared blank (Figure 1). The wells exposed to other test concentrations produced different shades of red, which indicated surviving metabolically active bacteria for at least some of the strains.

**Table 2.** A Comparison of BAG effects versus control.

		BOAT			Bactericidal test		Confocal laser scanning microscopy with LIVE/DEAD staining		
Test solution	*n*	Mean	±1 SD	*p* Value	Surviving strains/total no. strains	*p* Value	Ratio dead/total cell no.	±1 SD	*p* Value
Untreated bacterial strains (control)	11	2.884	0.11	–	11/11	–	0.190	0.01	–
Supernatant test									
BAG-S53P4 < 45 µm, 100 mg/mL, 2 days	11	2.704	0.49	0.30	11/11	–	0.176	0.12	0.85
BAG-S53P4 < 45 µm, 200 mg/mL, 2 days	11	2.402	0.96	0.15	11/11	–	0.746	0.35	0.07
BAG-S53P4 < 45 µm, 400 mg/mL, 2 days	11	0.789	0.72	<0.001	8/11	0.08	0.931	0.21	0.01
BAG-S53P4 < 45 µm, 100 mg/mL, with ciprofloxacin, 2 days	10	1.635	0.86	0.001	10/10	–	0.811	0.32	0.047
BAG-S53P4 < 45 µm, 200 mg/mL, with ciprofloxacin, 2 days	10	0.754	0.80	<0.001	10/10	–	0.836	0.11	0.002
BAG-S53P4 < 45 µm, 400 mg/mL, with ciprofloxacin, 2 days	10	0.208	0.09	<0.001	4/10	0.01	1.099	0.07	<0.001
BAG-S53P4 < 45 µm, 100 mg/mL, 14 days	11	1.208	1.07	0.001	11/11	–	0.686	0.13	0.008
BAG-S53P4 < 45 µm, 200 mg/mL, 14 days	11	0.173	0.05	<0.001	3/11	0.005	0.958	0.26	0.015
BAG-S53P4 < 45 µm, 400 mg/mL, 14 days	11	0.152	0.03	<0.001	0/11	0.001	1.086	0.05	<0.001
BAG-S53P4 < 45 µm, 100 mg/mL, with ciprofloxacin, 14 days	10	1.163	0.44	0.002	10/10	–	0.871	0.13	0.003
BAG-S53P4 < 45 µm, 200 mg/mL, with ciprofloxacin, 14 days	10	0.257	0.10	<0.001	2/10	0.008	1.099	0.12	0.001
BAG-S53P4 < 45 µm, 400 mg/mL, with ciprofloxacin, 14 days	10	0.207	0.11	<0.001	0/10	0.002	1.129	0.12	0.001
Granule test									
BAG-S53P4 < 45 µm, 100 mg/mL	11	N/A	N/A	N/A	0/11	0.001	1.046	0.11	0.001
pH-adjusted TSB									
TSB with 1% glucose/1% NaCl, pH 9.7	11	2.915	0.11	0.567	11/11	–	0.236	0.05	0.17
TSB with 1% glucose/1% NaCl, pH 10.7	11	0.749	0.80	<0.001	6/11	0.025	0.731	0.20	0.02
TSB with 1% glucose/1% NaCl, pH 11.7	11	0.129	0.02	<0.001	0/11	0.001	1.170	0.04	<0.001

Significance calculated by paired *t* test or McNemar asymptotic test.

BAG: bioactive glass; BOAT: biofilm-oriented antiseptics test; N/A: not applicable; SD: standard deviation; TSB: tryptic soy broth.

A pH-adjusted TSB solution was used as a control and shown to have a significant effect on the reduction of staphylococcal metabolic activity at pH 11.7 and 10.7, but not pH 9.7. All wells were completely blank with no visible shades of red colour only for the highest pH of 11.7.

The efficacy of BAG in the supernatant test was performed with and without ciprofloxacin. The tests showed an additional reduction of viable bacteria with ciprofloxacin only in concentrations of 100 mg/mL (*p* = 0.005) and 200 mg/mL primed for 48 h (*p* = 0.001). There was no additional effect of ciprofloxacin at any concentration of the supernatants primed for 14 days.

### Biofilm bactericidal test

In the granule test, all 11 strains of staphylococci biofilm were eradicated. In the supernatant tests, no bactericidal effects were recorded for the 48-h primed 100 and 200 mg/mL or the 14-day primed 100 mg/mL. In the supernatant test, only the 14-day primed 400 mg/mL concentration eradicated all strains (Table 2). For the remaining concentrations and durations, there was a significant bactericidal effect, but not complete eradication for all strains (Table 2). There was an additional effect of ciprofloxacin in the bactericidal test only with 400 mg/mL, 48-h primed (*p* = 0.046). Ciprofloxacin showed no additional bactericidal effect for the other concentrations.

### Confocal laser scanning microscopy (CLSM)

**Figure 2.**
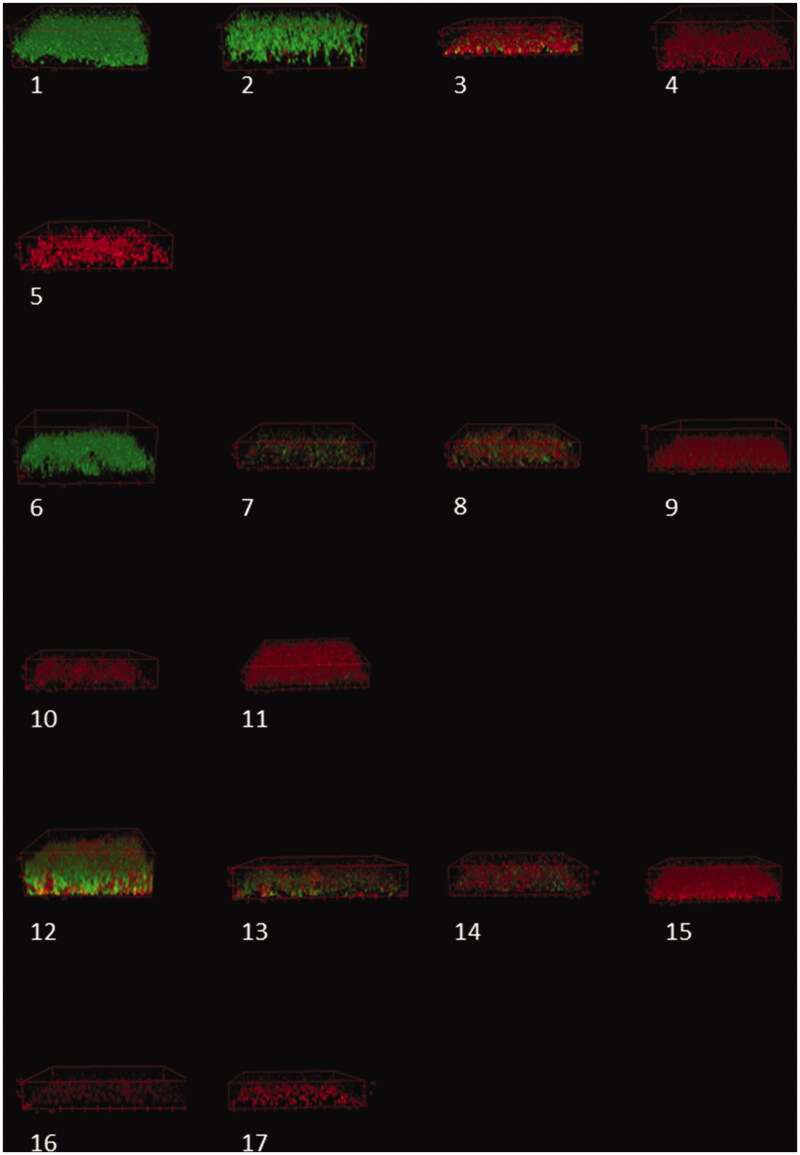
Confocal laser scanning microscopy (CLSM) stacks of *Staphylococcus aureus* biofilm. 1) Control; pH-adjusted TSB: 2) pH 9.7; 3) pH 10.7; 4) pH 11.7; 5) Granule test; Supernatant test, 2-day exposure: 6) 100 mg/mL; 7) 100 mg/mL with ciprofloxacin; 8) 200 mg/mL; 9) 200 mg/mL with ciprofloxacin; 10) 400 mg/mL; 11) 400 mg/mL with ciprofloxacin; Supernatant test, 14-day exposure: 12) 100 mg/mL; 13) 100 mg/mL with ciprofloxacin; 14) 200 mg/mL; 15) 200 mg/mL with ciprofloxacin; 16) 400 mg/mL; 17) 400 mg/mL with ciprofloxacin. The units are in µm.

In the granule test, the ratio of compromised cells to the total number of cells was close to 1, indicating that all bacteria were dead or dying (Table 2; Figure 2). In the supernatant test, there was a reduction in the 48-h primed solutions only for the concentration of 400 mg/mL (Table 2; Figure 2). Some viable biofilm bacteria were still observed. There was a significant reduction in viable cells for all concentrations primed for 14 days. The ratio of dead to total number of cells was close to 1 only for the concentrations of 200 and 400 mg/mL (Table 2). CLSM showed an additional effect of ciprofloxacin only in the group with 100 mg/mL (48 h primed).

### Summary of results

- BAG granules (100 mg/mL) eradicated *Staphylococci aureus* in planktonic form.
- BAG supernatant (200 mg/mL) eradicated *Staphylococci aureus* in planktonic form.
- BAG granules (100 mg/mL) eradicated *Staphylococci aureus* in biofilm in the biofilm bactericidal test.
- BAG supernatant (400 mg/mL) primed for 14 days eradicated *Staphylococci aureus* in biofilm in the biofilm bactericidal test.
- BOAT tests showed that there was a reduction in metabolic activity for all concentrations after 14-day priming, but only for the 400 mg/mL concentration after 48 h priming.
- BAG granules (100 mg/mL) showed reduction in viable bacteria in biofilm on CLSM.
- BAG supernatant showed reduction in viable bacteria in biofilm for all concentrations primed for 14 days, and for 400 mg/mL primed for 48 h on CLSM.
- Ciprofloxacin had an additional effect only with a 48 h priming time.

## Discussion

Several studies have indicated a difference in the antimicrobial sensitivity of laboratory and clinical strains (35–37). The strength of the present study is the relatively large number of clinically relevant strains compared to previous studies, and the use of three separate test systems. All three tests showed significant antibacterial properties exhibited by BAG against staphylococci in biofilm and in planktonic states. Our results show that primed BAG granules in direct contact with biofilm eradicate all viable bacteria even at the lowest concentration and shortest priming time. This indicates that BAG is a potent anti-biofilm substance.

Previous studies have indicated that small granules have a significantly stronger antimicrobial effect, and reach a higher pH in solution, compared with larger granules, supposedly because of a greater surface area (16). Therefore we used the smallest available granules (BAG-S53P4, <45 µm). Our results support the use of small granule size to rapidly gain full antimicrobial effect.

It is of great interest to know if the supernatant fluid primed with granules retains its antimicrobial and anti-biofilm properties after physical removal of the granules. To our knowledge, this has not been previously investigated for staphylococcal biofilms. The results show that the surrounding fluid itself assumes anti-biofilm properties after long enough priming, even when isolated from the physical granules. Complex bone cavities, such as the mastoid cell system, are often difficult to surgically pack, and may have pockets that remain incompletely filled with granules despite efforts to pack them completely. Therefore it is important to know whether the supernatant has the same effect as granules. When the supernatant from the lowest concentration and 48-h priming time was tested, there was no reduction in viable biofilm (Table 2). The same concentration with 14-day priming resulted in reduction in viable biofilm bacteria when using CLSM and BOAT. However, only the supernatant from the concentration of 14-day primed 400 mg/mL BAG eradicated all the biofilm bacteria strains in all three test systems. This shows that the anti-biofilm effect on biofilm-associated staphylococci is dependent on concentration and priming time and that it could be beneficial to use pre-primed saline solution. This can be of importance when packing chronically infected bone cavities with biofilm-associated staphylococci.

Ciprofloxacin, which has good penetration into biofilms (38), was evaluated regarding possible additional antimicrobial effect when combined with BAG. CLSM and BOAT showed a significant reduction in viable bacteria in biofilm by combining BAG and ciprofloxacin for the two lowest concentrations primed for 48 h, compared with BAG only (Table 2). However, the combination did not show complete bactericidal effects. This indicates that the addition of ciprofloxacin to a BAG treatment could render some additional antimicrobial effect, which could be clinically beneficial during the first days of treatment before BAG reaches its maximal effect on biofilm-associated bacteria.

The antibacterial properties of BAG have been attributed to its high pH and osmotic effects caused by the non-physiological concentration of dissolved ions (39). Pre-adjusted pH-buffered (NaOH) TSB broth was used as a control to compare with BAG at similar pH. The most alkaline control was set to pH 11.7, the same as achieved by BAG primed for 14 days at 400 mg/mL. At this pH level, there was a significant reduction in amount of viable bacteria in biofilm in all three test systems. It was also the only pH that eradicated all bacteria in the bactericidal biofilm test. Previous studies have shown that *S. aureus* can survive up to a pH of 9.83 (40,41). This explains why the TSB control broth (pH 9.7) and BAG supernatant (100 mg/mL, primed for 48 h) (pH 9.9) showed no reduction in the amount of viable biofilm-associated bacteria. When the pH of the TSB control broth was raised to 10.7, five out of eleven strains were eradicated. Supernatant from BAG 400 mg/mL 48-h priming resulted in a pH of 10.6, and three out of eleven strains were eradicated. This finding is in line with previous studies (42) and indicates that high pH is an important factor against biofilm-associated bacteria.

These *in vitro* results are promising, but several factors *in vivo* can affect the clinical response. If the environment around the biofilm does not achieve bactericidal pH, *S. aureus* can upregulate a Na^+^/H^+^ antiporter system in response to a sudden alkaline shock environment (43). Although further clinical studies are required to explore the antimicrobial effects on biofilm *in vivo*, this *in vitro* study indicates that both supernatant and granules of BAG can give bactericidal anti-planktonic and anti-biofilm properties. This reduces the likelihood of recurrent infections after incomplete filling of the bone cavity with BAG (20,44). The tested granules are smaller than the granules typically used in bone packing. Our study suggests that the risk of recurrent infections could possibly be reduced by using small granules, <45 µm, which proved to give a more rapid and higher rise in pH. We also showed that the bactericidal effect of BAG is dependent on exposure time and concentration. When BAG has reached its full antimicrobial effect it is as efficient as ciprofloxacin *in vitro* and could reduce the use of conventional antibiotics where *S. aureus* biofilm is suspected. Our results also suggest that short-term use of antibiotics at the beginning of treatment may be beneficial.

We conclude that BAG-S53P4, <45 µm, has powerful antimicrobial effects on *S. aureus* in both planktonic and biofilm states. Duration of exposure and concentration are important factors for achieving maximal effect on biofilm-associated bacteria. Ciprofloxacin may have an additional effect during the first days of treatment.
